# Diffusion-weighted imaging of rectal cancer on repeatability and cancer characterization: an effect of b-value distribution study

**DOI:** 10.1186/s40644-018-0177-1

**Published:** 2018-11-15

**Authors:** Luguang Chen, Fu Shen, Zhihui Li, Haidi Lu, Yukun Chen, Zhen Wang, Jianping Lu

**Affiliations:** Department of Radiology, Changhai Hospital of Shanghai, The Second Military Medical University, No.168 Changhai Road, Shanghai, 200433 China

**Keywords:** Rectal cancer, Diffusion-weighted imaging, Apparent diffusion coefficient, T stage, B-value

## Abstract

**Background:**

To explore the effect of b-value distributions on the repeatability and diagnostic performance of the ADC value in rectal cancer patients using multiple b-values and mono-exponential model diffusion-weighted imaging (DWI).

**Methods:**

Thirty-two preoperative rectal cancer patients, without receiving neoadjuvant therapy, were scanned on a 3 Tesla magnetic resonance imaging scanner using DWI with 10 b-values ranging from 0 to 2000 s/mm^2^. The apparent diffusion coefficient (ADC) value was calculated using a mono-exponential model and 31 b-value combinations consisting of 2 to 10 b-values were explored. Regions of interest with the maximum cross-sectional tumour size were outlined on the ADC map by two independent observers. Intraclass correlation coefficients (ICC), coefficient of variation (CV), and Bland-Altman plots between the two observers were calculated and evaluated to determine repeatability. Areas under receiver operating characteristic curves (AUCs) were evaluated for rectal cancer characterization. Correlations between the mean ADC values and T stage were assessed using the Spearman correlation coefficient (ρ). α (= ICC + AUC + |ρ|- CV - |bias|) was defined and used to assess the optimal b-value distribution.

**Results:**

Postoperative pathology tests revealed 4 patients with T1, 10 patients with T2, and 18 patients with T3 stages. There were no significant difference in age and sex between the two groups (T1–2 vs. T3). Excellent reproducibility was observed for ADC values between two observers with ICC and CV values ranging from 0.920 to 0.998, and 1.475 to 5.568%, respectively. The mean percent difference and ρ between the paired measurements was ranged from − 2.7 to 1.2% and from − 0.759 to − 0.407, respectively. The b-value combinations with the top three α values were b(0, 1000 s/mm^2^), b(500, 1500, 2000 s/mm^2^) and b(100, 1000, 1500 s/mm^2^) for α = 2.581, 2.571 and 2.569, respectively.

**Conclusions:**

The number of b-values and their distributions influenced the repeatability of the ADC values and their diagnostic performance. The optimal b-value combination was 0 and 1000 s/mm^2^ for DWI examination of rectal cancer patients.

**Electronic supplementary material:**

The online version of this article (10.1186/s40644-018-0177-1) contains supplementary material, which is available to authorized users.

## Background

Colorectal cancer is the third most commonly diagnosed cancer worldwide, and is the fifth most common cancer in China, with 376,300 new cases and 191,000 deaths in 2015, and with the incidence is steadily increasing [1, 2]. Rectal cancer accounts for 30–35% of those cases [3], which are generally adenocarcinomas, and are managed with a combination of surgery, chemotherapy and radiation therapy [4]. Therefore, early and accurate preoperative staging is critical for decision-making regarding treatment in clinical practice.

Diffusion-weighted imaging (DWI), as a functional magnetic resonance imaging (MRI), has been used to evaluate the Brownian movement of water molecules in tissues in vivo. The apparent diffusion coefficient (ADC), which is derived from diffusion-weighted images, has been used as a quantitative parameter for assessing the water diffusion through tissue. It has been shown that ADC values have a negative correlation with tissue cellularity [5, 6]. The increased cellularity and structural distortion of tumour cells in the extracellular space result in reduced ADC values [7]. Single-shot echo-planar imaging (SS-EPI) is one of the most commonly used DWI techniques in a routine examination. The ADC value has been used for the diagnosis of rectal cancer and for evaluating the therapeutic effect of neoadjuvant chemoradiotherapy, while reflecting some histological characteristics of the lesions [8, 9].

Early DWI techniques were mainly focused on using two b-values which limited the analysis of diffusion-weighted images to the simplest mono-exponential model. With the rapid improvement of MRI technology, state-of-the-art MRI scanners have the ability to perform DWI in the body using multiple b-values which may provide more information for lesion characterization. For a successful multiple b-value DWI experiment, it is important to optimize the combination of b-values; at least two or three b-values (such as a lower b-value < 100 s/mm^2^, an intermediate b-value 400–500 s/mm^2^, and a higher b-value between 500 to 1000 s/mm^2^) should be used for clinical purposes [10, 11]. Previous studies have explored the correlation between the mean ADC values and the diagnosis of rectal cancer using DWI with two or more b-values, ranging from 0 to 800–1000 s/mm^2^ [12–18]. However, the effect of the b-value distribution on the diagnostic performance has not yet been explored. An accurate estimation of diffusion properties with high repeatability plays a vital role in the use of DWI for non-invasive characterization of rectal cancer. Moreover, the use of an optimized b-value combination could be an important step in the optimization of rectal DWI. We hypothesized that the optimized b-value combination may offer an improved diagnostic performance and reproducibility in assessing rectal lesions.

Therefore, the aim of the present study was to explore the effect of b-value distributions on the repeatability and diagnostic performance of the ADC value in rectal cancer patients using the multiple b-value and mono-exponential DWI model.

## Methods

### Subjects

This study was approved by the local institutional review board and all subjects signed written informed consent. Between March 2017 and September 2017, 35 patients with rectal lesions identified via colonoscopy were consecutively recruited in this study. All subjects underwent a multiple b-value DWI examination and had a postoperative pathology test. Patients who received chemotherapy or radiotherapy before and after MRI, had contraindications to MRI, or had poor image quality were excluded. One patient received CRT after MRI, one patient had claustrophobia and one patient had poor image quality due to motion artefacts. A total of 32 patients were included in the final analysis, who were confirmed via pathology as rectal cancer with moderately differentiated adenocarcinoma. All patients were divided into two groups according to the tumour T stages based on the postoperative pathology reports: (1) T1 and T2 stages; and (2) T3 and T4 stages. The criteria for T3 subcategories were defined as follows: T3a, tumor extends < 1 mm beyond muscularis propria; T3b, tumor extends ≥ 1-5 mm beyond muscularis propria; T3c, tumor extends > 5-15 mm beyond muscularis propria; T3d, tumor extends > 15 mm beyond muscularis propria [17].

### Magnetic resonance imaging

All imaging was performed on a 3 Tesla MRI scanner (MAGNETOM Skyra, Siemens Healthcare GmbH, Erlangen, Germany) using a pelvic phased-array coil. Each subject fasted for 4 h before scanning, and antiperistaltic drugs were not used. Axial SS-EPI DWI with 10 b-values was used and the main imaging parameters were presented as follows: repetition time/echo time (TR/TE) = 6300/89 ms, field of view (FOV) = 380 × 380 mm^2^, matrix = 150 × 150, slices = 20, slice thickness = 5 mm, gap = 1 mm, acceleration factor = 2, bandwidth = 2084 Hz/pixel. b-values (number of signal averages) = 0 (1), 50 (1), 100 (1), 150 (1), 200 (1), 250 (2), 500 (2), 1000 (2), 1500 (3), and 2000 (3) s/mm^2^, and acquisition time = 5 min 47 s. In addition, axial high-resolution T2-weighted turbo spin echo images were acquired with the following parameters: TR/TE = 4000/108 ms, FOV = 180 × 180 mm^2^, matrix = 320 × 320, slice thickness = 3 mm, gap = 0 mm, acceleration factor = 3, echo train length = 16 and acquisition time = 4 min 10 s. The time interval between the MRI and surgery was 6.3 ± 5.9 days, ranging from 2 to 30 days.

### Image analysis

Data obtained from multiple b-value DWI was sent to an advanced workstation and all images were independently evaluated by two experienced observers (with 8 and 5 years of experience in pelvic radiology, respectively) using the prototype post-processing software (Body Diffusion Toolbox, Siemens healthcare GmbH, Germany). DWI data with different b-value combinations was fitted using the mono-exponential model, *S(b)* = *S(0) e* ^–b*ADC^, where *S(b)* and *S(0)* indicate the signal intensity at a b-value > 0 and = 0 s/mm^2^, respectively.

According to the combination principle, in total there were 1013 possible b-value combinations, ${C}_{10}^2$(=45), ${C}_{10}^3$(=120), ${C}_{10}^4$(=210), ${C}_{10}^5$(=252), ${C}_{10}^6$(=210), ${C}_{10}^7$(=120), ${C}_{10}^8$(=45), ${C}_{10}^9$(=10), and ${C}_{10}^{10}$(=1) for the combinations of 2, 3, 4, 5, 6, 7, 8, 9, and 10 b-values, respectively. However, in the current study, 31 representative combinations of 2, 3, 4, 5, 6, 7, 8, 9, and 10 b-values were selected to evaluate repeatability and diagnostic performance based on the existing reports.

**Fig. 1:**
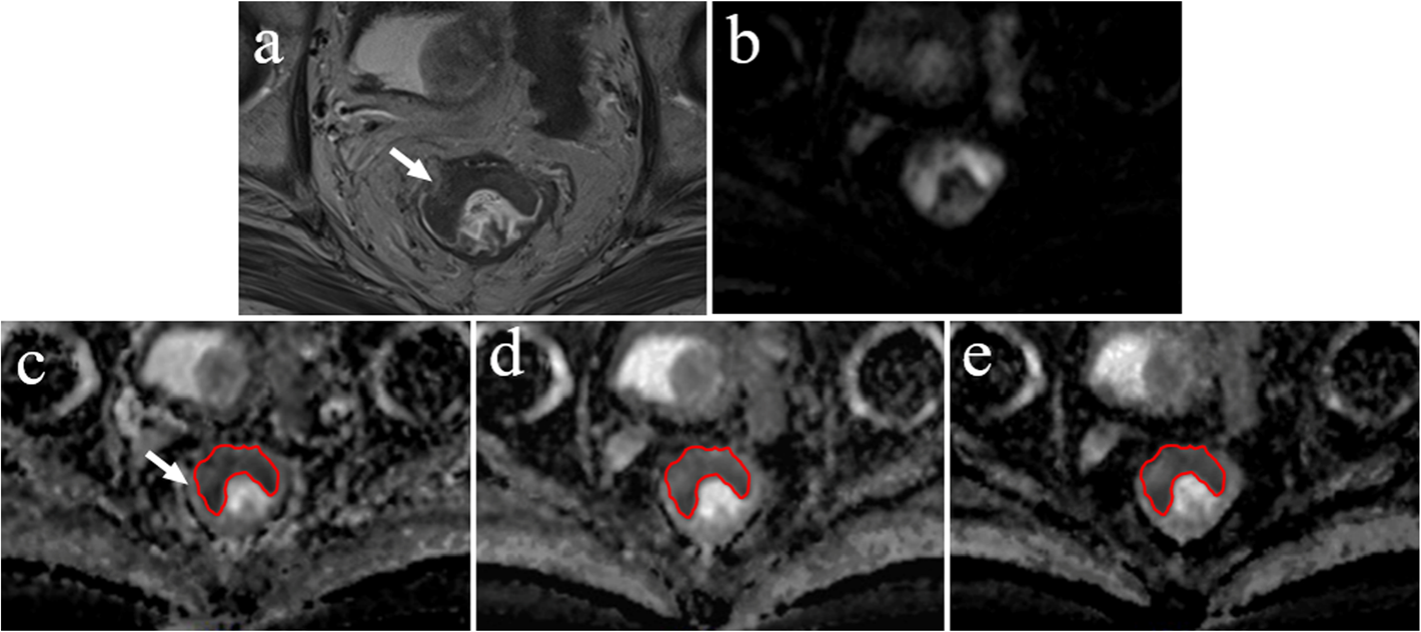
Example images for delineating ROI of rectal lesion. **a** T2W image. **b** Diffusion-weighted image at b = 1000 s/mm^2^. **c** ADC map derived from a b-value combination of 0, 1000 s/mm^2^. **d** ADC map derived from a b-value combination of 100, 1000, 1500 s/mm^2^. **e** ADC map derived from a b-value combination of 500, 1500, 2000 s/mm^2^

Each region of interest (ROI) with a maximum cross-sectional tumour size was outlined on the ADC map so that the T2 shine-through effect could be effectively avoided, which is helpful for displaying clear border between the tumour and normal tissue [19]. Additionally, DWI and T2W images were used as a reference to delineate the tumour on ADC images (Fig. 1). The mean ADC values of the lesions were measured using the single-slice ROI method, which is briefly described in the following steps: select the maximum slice of the lesion, delineate the entire range of the lesion, measure three times and then calculate the average [19–21]. In addition, the areas of ROI were also recorded.

To ensure the images had adequate signal-to-noise ratios (SNR) for quantification, the SNR of diffusion-weighted images at b-value = 2000 s/mm^2^ for each patient was calculated as the ratio of signal intensity of the ROI in rectal cancer divided by the mean standard deviation of four same-sized ROIs (25 voxels) distributed in the background that near the anterior abdominal wall on the same slice, without imaging artefacts.

To explore an optimized b-value combination, a comprehensive analysis of the factors for reliability and diagnostic performance was performed. We introduced a new parameter, called α, to characterize these factors: α = ICC + AUC + |ρ|- CV - |bias|, where ICC is the intraclass coefficient; AUC is the area under the receiver operating characteristic curve; ρ indicates the Spearman correlation coefficient; CV is the coefficient of variability; and bias indicates the mean of the percent difference for the paired measurements. Therefore, the larger α is, the more optimized the b-value combination is.

### Statistical analysis

SPSS software (version 16.0, Inc., Chicago, IL, USA) for Microsoft Windows was used for statistical analysis. Data are expressed as the mean ± standard deviation. The difference in age between the two groups was assessed using a paired t-test, and a Chi-square test was used to evaluate the group difference in sex. To evaluate the interobserver variability, ICC, CV, and Bland-Altman plots were performed to assess the ADC measurements with different b-value combinations. ICC values < 0.4 indicates poor agreement; 0.4–0.75 indicates good agreement and > 0.75 indicates excellent agreement [22]. In addition, the correlations between the mean ADC values of the two observers and T stages were analysed using Spearman’s rank correlation test. ROC analysis were performed to differentiate T3 from T1–2 stages based on whether the tumour invaded into the fat tissue surrounding the rectum. A *p*-value < 0.05 was inferred to statistically significance.

## Results

### Characteristics of the patients

Among the 32 patients with rectal cancer, 16 were males and 16 were females, with a mean age of 59.1 ± 8.9 years (range 35–78). None of them had received neoadjuvant therapy. Histopathological staging revealed 4 lesions in T1, 10 lesions in T2, 18 lesions in T3 (5 lesions in T3a, 9 lesions in T3b, 3 lesions in T3c, and 1 lesion in T3d), and none in the T4 stage. All patients were confirmed as moderately differentiated adenocarcinoma. Each patient had a single lesion. There were 10 tumours located in the lower rectum, 12 tumours located in the middle rectum, and 10 tumours located in the superior rectum. There was no significant difference in age and sex between the two groups according to the tumor T stages (Table 1).

**Table 1:** Characteristics of the patients with rectal cancer

Group	*n*	Sex		Age (Years,$\overline{x}$±*s*)
		Male	Female	
T1 + 2	14	7	7	58.5 ± 6.5
T3	18	9	9	59.6 ± 10.2
Statistical value		0.127^a^		−38.086^b^
*p*-value		0.063		< 0.0001

^a^ *χ*^*2*^ – value

^b^ *t* – value

### B-value distribution

Thirty-one b-value combinations were evaluated in the final analysis. The number and b-value distributions are listed in Table 2. They are: 9 combinations with 2 b-values, 6 combinations with 3 b-values, 5 combinations with 4 b-values, 5 combinations with 5 b-values, 2 combinations with 6 b-values, 1 combination with 7 b-values, 1 combination with 8 b-values, 1 combination with 9 b-values, and 1 combination with all b-values.

**Table 2:** Repeatability and diagnostic performance of the ADC parameter

No	b-value distribution	ICC (95% CI)	CV (%)	bias (%, ±1.96SD)	AUC (95% CI)	ρ (95% CI)	α
2	0,1000	0.984(0.967–0.992)	3.840	−1.1 (− 11.0–8.9)	0.901 (0.743–0.978)	− 0.745(− 0.868 - -0.535)	2.581
2	0,1500	0.970(0.938–0.985)	4.063	0.8 (− 10.1–11.6)	0.829 (0.655–0.939)	− 0.629(− 0.802 - -0.359)	2.379
2	0,2000	0.968(0.933–0.984)	3.729	1.2 (− 8.7–11.2)	0.837 (0.664–0.943)	− 0.629(− 0.802 - -0.359)	2.385
2	50,1000	0.978(0.955–0.989)	3.333	0.6 (− 8.7–10.0)	0.877 (0.713–0.966)	− 0.710(− 0.849 - -0.480)	2.526
2	50,1500	0.977(0.954–0.989)	3.531	− 0.1 (− 10.2–10.0)	0.813 (0.637–0.929)	− 0.618(− 0.795 - -0.343)	2.372
2	100,1000	0.985(0.970–0.993)	3.514	− 1.8 (− 10.4–6.8)	0.730 (0.545–0.871)	−0.494(− 0.719 - -0.175)	2.156
2	100,2000	0.984(0.966–0.992)	3.762	0.4 (− 9.7–10.5)	0.837 (0.664–0.943)	− 0.598(− 0.783 - -0.314)	2.377
2	200,1000	0.976(0.952–0.989)	3.582	−0.9 (− 10.1–8.3)	0.734 (0.549–0.874)	−0.462(− 0.698 - -0.135)	2.127
2	200,2000	0.988(0.975–0.994)	2.991	0.4 (− 8.5–9.2)	0.766(0.583–0.897)	− 0.511(− 0.730 - -0.197)	2.231
3	0,100,1000	0.990(0.979–0.995)	2.407	−0.6 (− 7.3–6.1)	0.825(0.651–0.936)	−0.635(− 0.805 - -0.368)	2.420
3	0,200,2000	0.980(0.959–0.990)	2.963	− 1.1 (− 9.0–6.7)	0.766(0.583–0.897)	−0.525(− 0.739 - -0.216)	2.230
3	50,1000,2000	0.989(0.978–0.995)	2.857	0.4 (− 7.7–8.6)	0.865(0.698–0.959)	−0.644(− 0.810 - -0.380)	2.465
3	100,1000,1500	0.958(0.914–0.979)	5.568	− 1.7 (− 16.1–12.7)	0.933(0.785–0.991)	−0.751(− 0.871 - -0.544)	2.569
3	200,500,1500	0.966(0.929–0.983)	3.613	−2.2 (− 11.3–6.9)	0.802(0.623–0.921)	−0.572(− 0.767 - -0.278)	2.282
3	500,1500,2000	0.922(0.840–0.962)	4.374	−0.3 (− 11.2–10.7)	0.937(0.791–0.992)	−0.759(− 0.876 - -0.558)	2.571
4	0,100,1000,1500	0.989(0.977–0.995)	3.077	−2.6 (− 10.4–5.2)	0.750(0.549–0.874)	−0.496(− 0.706 - − 0.149)	2.178
4	0,200,1000,2000	0.990(0.980–0.995)	2.143	-0.1 (− 6.0–5.8)	0.833(0.660–0.941)	−0.595(− 0.781 - -0.310)	2.396
4	0,500,1000,1500	0.983(0.965–0.992)	3.703	− 1.4 (− 10.0–7.1)	0.829(0.655–0.939)	−0.574(− 0.769 - -0.282)	2.335
4	100,200,500,1000	0.994(0.988–0.997)	2.554	−2.7 (− 7.9–2.6)	0.804(0.625–0.922)	−0.567(− 0.765 - -0.272)	2.312
4	500,1000,1500, 2000	0.920(0.835–0.961)	4.892	− 1.9 (− 13.5–9.7)	0.938(0.793–0.993)	−0.748(− 0.870 - -0.540)	2.538
5	0,50,200,500,1000	0.998(0.996–0.999)	1.475	−1.6 (− 4.5–1.2)	0.714(0.528–0.859)	−0.407(− 0.662 - -0.068)	2.088
5	0,200,500,1000,1500	0.995(0.990–0.998)	1.933	−1.6 (− 6.0–2.9)	0.774(0.592–0.902)	−0.496(− 0.720 - -0.179)	2.230
5	0,500,1000,1500,2000	0.984(0.968–0.992)	3.349	−1.4 (− 9.7–6.8)	0.871(0.705–0.963)	−0.664(− 0.822 - -0.410)	2.472
5	50,200,500,1500,2000	0.982(0.962–0.991)	2.735	−0.9 (− 7.9–6.0)	0.865(0.698–0.959)	−0.664(− 0.810 - -0.380)	2.475
5	100,200,500,1000,2000	0.990(0.979–0.995)	2.263	−1.6 (− 6.8–3.6)	0.861(0.693–0.957)	−0.626(− 0.800 - -0.355)	2.438
6	0,100,200,500,1000,1500	0.996(0.992–0.998)	1.784	−1.4 (− 5.6–2.8)	0.829 (0.655–0.939)	−0.609(− 0.790 - -0.331)	2.402
6	50,200,500,1000,1500,2000	0.988(0.976–0.994)	2.520	−1.7 (− 7.6–4.3)	0.825(0.651–0.936)	−0.589(− 0.778 - -0.302)	2.360
7	0,100,200,500,1000,1500,2000	0.993(0.985–0.996)	2.137	−1.5 (−6.4–3.5)	0.821(0.646–0.934)	−0.597(− 0.783 - -0.314)	2.375
8	0,50,100,200,250,500,1000,1500	0.995(0.991–0.998)	1.966	−1.6 (− 6.2–3.0)	0.817(0.641–0.931)	−0.597(− 0.783 - -0.314)	2.373
9	0,50,100,150,200,500,1000,1500,2000	0.988(0.975–0.994)	2.811	−1.9 (−8.6–4.8)	0.857(0.688–0.955)	−0.618(− 0.795 - -0.343)	2.416
10	All b values	0.991(0.981–0.996)	2.566	−2.0 (− 7.7–3.6)	0.774(0.592–0.902)	−0.471(− 0.704 - -0.146)	2.190

*ICC* intraclass correlation coefficient, *SD* standard deviation, *CI* confidence interval, *CV* coefficient of variation, *AUC* area under receiver operating characteristic curve, ρ: Spearman correlation coefficient, α: = ICC + AUC + |ρ|- CV - |bias|

### SNR and ROI size

The mean SNR of diffusion-weighted images at a b-value of 2000 s/mm^2^ was 92.74 ± 17.14. No significant difference was observed between the two observers in ROI size delineation (303.0 ± 167.0 mm^2^ vs. 305.4 ± 175.9 mm^2^, *p* = 0.724).

### Reproducibility of ADC measurements

**Fig. 2:**
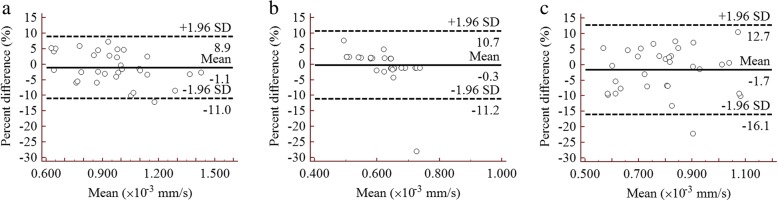
Bland-Altman plots show good agreement of the ADC measurements between two observers for three representative b-value combinations. **a** a b-value combination of 0, 1000 s/mm^2^. **b** a b-value combination of 100, 1000, 1500 s/mm^2^. **c** a b-value combination of 500, 1500, 2000 s/mm^2^

The statistical results of repeated ADC measurements are presented in Table 2. There was an excellent reproducibility for ADC values between two observers with ICC and CV values ranging from 0.920 to 0.998, and 1.475 to 5.568%, respectively. In addition, the mean percent difference between the paired measurements was relatively small, with a range of − 2.7 to 1.2%. Besides, narrow intervals were observed for those measurements using Bland-Altman plots in three represented b-value combinations (Fig. 2).

### Correlation between mean ADC values and T stages, ROC analysis

**Fig. 3:**
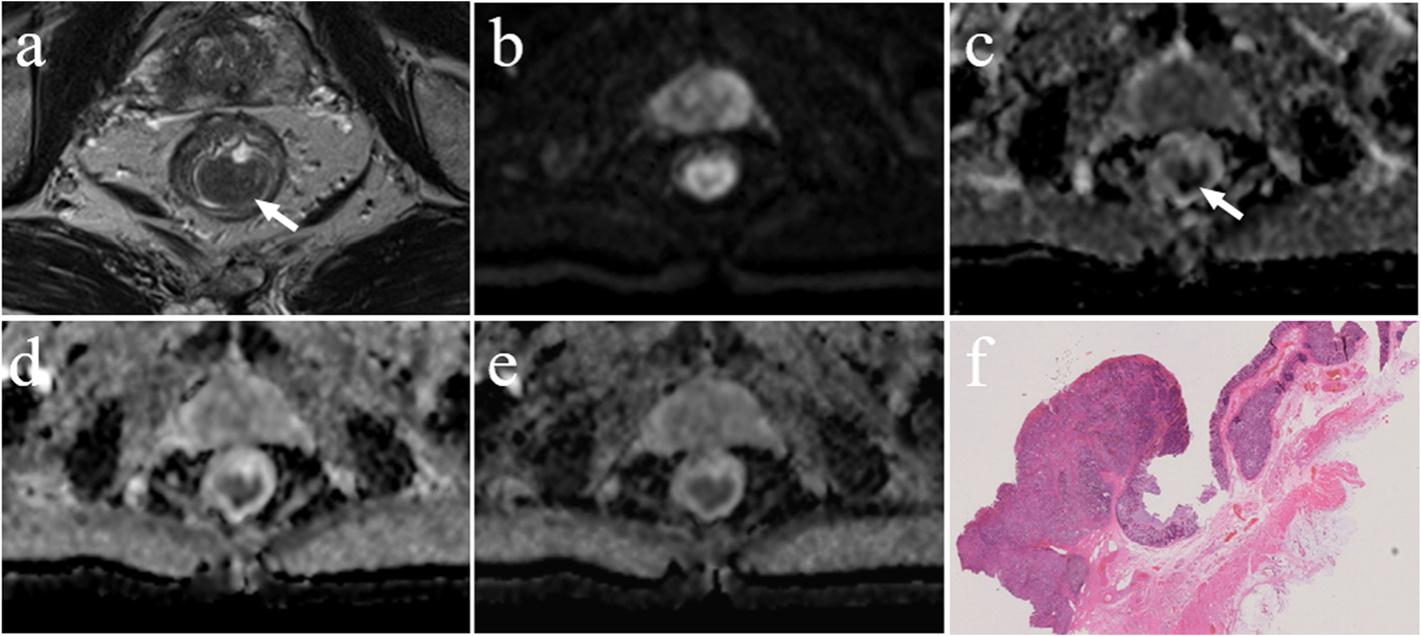
A 52-year-old female with moderately differentiated adenocarcinoma (T1 stage). **a** Axial high resolution T2WI shows the tumour of the posterior rectal wall (arrow). **b** Diffusion-weighted image at b = 1000 s/mm^2^. **c**-**e** The ADC map shows the lesion with a low-signal-intensity (arrow) at b = 0, 1000 s/mm^2^, the mean ADC value of the lesion is 1.409 × 10^− 3^ mm^2^/s (**c**). 100, 1000, 1500 s/mm^2^, the mean ADC value is 1.042 × 10^− 3^ mm^2^/s (**d**). 500, 1500, 2000 s/mm^2^, the mean ADC value is 0.675 × 10^− 3^ mm^2^/s (**e**). **f** Postoperative pathology (haematoxylin and eosin, × 1). Tumour cells invaded the submucosa

**Fig. 4:**
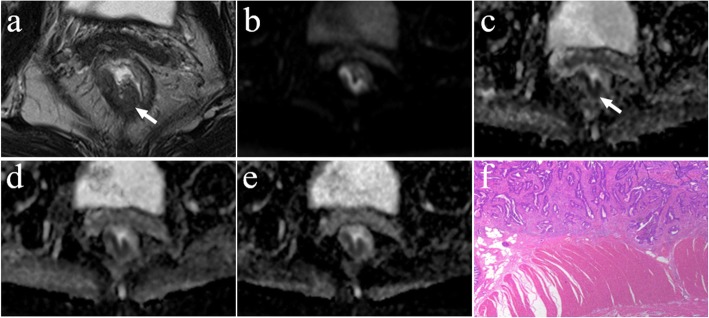
A 58-year-old male with moderately differentiated adenocarcinoma (T2 stage). **a** Axial high resolution T2WI shows the tumour of the posterior rectal wall (arrow). **b** Diffusion-weighted image at b = 1000 s/mm^2^. **c**-**e** The ADC map shows the lesion with a low-signal-intensity (arrow) at b = 0, 1000 s/mm^2^, the mean ADC value of the lesion is 0.999 × 10^− 3^ mm^2^/s (**c**). 100, 1000, 1500 s/mm^2^, the mean ADC value is 0.832 × 10^− 3^ mm^2^/s (**d**). 500, 1500, 2000 s/mm^2^, the mean ADC value is 0.647 × 10^− 3^ mm^2^/s (**e**). **f** Postoperative pathology (haematoxylin and eosin, × 25). Tumour tissue showed papillary and mesh-like alignment. Tumour cells had a cubic and column-like shape with big, atypical, and deeply stained nuclei, which invaded the muscularis propria layer but did not extend beyond it

**Fig. 5:**
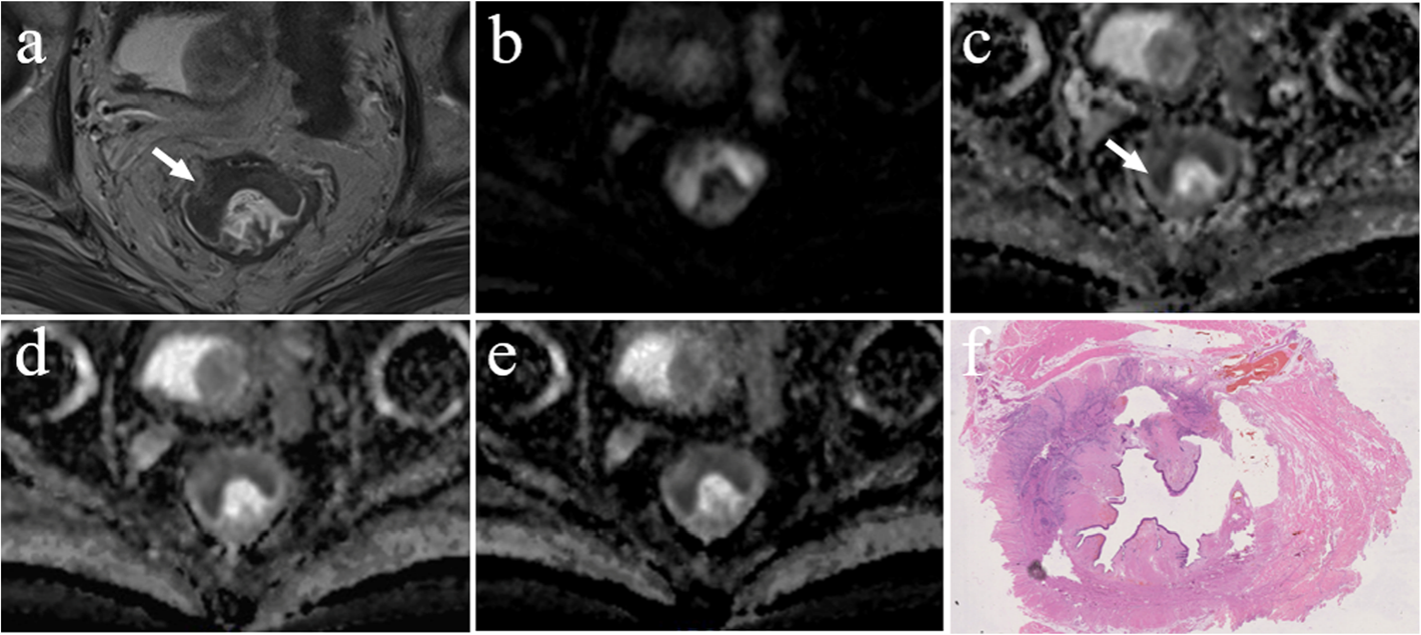
A 59-year-old male with moderately differentiated adenocarcinoma (T3 stage). **a** Axial high resolution T2WI shows abnormal signals on the front of the intestinal wall. The muscularis propria displayed discontinuity and marginal haziness with a low-signal-intensity (arrow). **b** Diffusion-weighted image at b = 1000 s/mm^2^. **c**-**e** The ADC map shows the lesion with a low-signal-intensity (arrow) at b = 0, 1000 s/mm^2^, the mean ADC value of the lesion is 0.737 × 10^− 3^ mm^2^/s (**c**). 100, 1000, 1500 s/mm^2^, the mean ADC value is 0.597 × 10^− 3^ mm^2^/s (**d**). 500, 1500, 2000 s/mm^2^, the mean ADC value is 0.513 × 10^− 3^ mm^2^/s (**e**). **f** Postoperative pathology (haematoxylin and eosin, macro sections, × 1). Tumour tissue shows the muscularis propria is completely disrupted and tumour extension is into the mesorectum

**Fig. 6:**
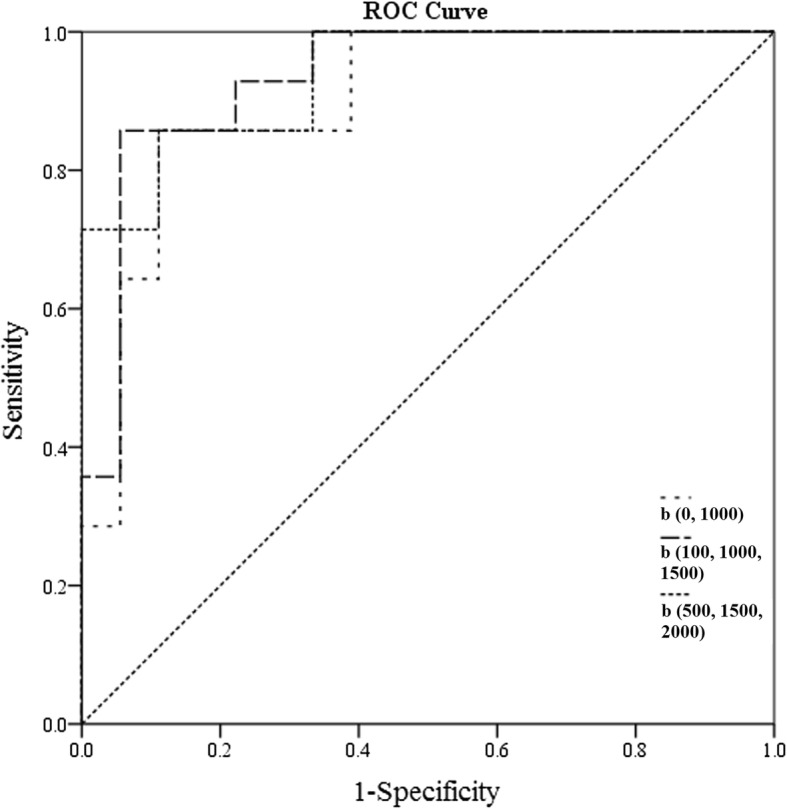
Receiver operating characteristic (ROC) curves of the mean ADC for discriminating rectal cancer between T stages 1 + 2 and 3 for the representative b-value combinations. Areas under receiver operating characteristic curves are 0.901 (0.743–0.978), 0.937(0.791–0.992), and 0.933(0.785–0.991) for b(0, 100), b(100, 1000, 1500), and b(500, 1500, 2000) combinations, respectively

There was a significant negative correlation between the mean ADC values and T stages for patients with rectal cancer in all b-value combinations. The Spearman correlation coefficients for those combinations ranged from − 0.759 to − 0.407. The results of the ROC analysis are shown in Table 2 and Additional file 1: Table S1. Overall, the AUC values ranged between 0.714 and 0.938 for evaluating the mean ADC values and T stages. Representative examples of the images in rectal cancer patients with T1, T2 and T3 stages are shown in Figs. 3, 4, 5, respectively. Figure 6 shows the ROC curves of the mean ADC for discriminating rectal cancer between T1–2 and T3 stages for the representative b-value combinations.

### Optimized b-value distribution

To obtain the optimized b-value distribution, α was defined and calculated according to the above-mentioned formula, in which ICC, CV, bias, AUC and ρ values were taken into consideration. The results of the α values are also presented in Table 2. b-value combinations with the top three α values are b(0, 1000 s/mm^2^), b(500, 1500, 2000 s/mm^2^) and b(100, 1000, 1500 s/mm^2^) for α = 2.581, 2.571 and 2.569, respectively. Therefore, the optimal b-value combination is b-value = 0 and 1000 s/mm^2^, which has the highest α = 2.581. The optimal b-value combination makes it possible to provide more information on distinguishing the T3 stage from the T2 stage. The ADC cut-off threshold for the recommended b-value combination (0 and 1000 s/mm^2^) is 0.979 × 10^− 3^ mm^2^/s.

## Discussion

The present study evaluated how the optimized b-value distribution contributes to the improved repeatability of the measurements and whether a sufficient number of b-values with similar diagnostic performances is achieved. Meanwhile, the ADC measurement in the preoperative diagnosis of rectal cancer was explored.

In recent years, diffusion-weighted magnetic resonance imaging has served as a non-invasive technique and has been used to measure the Brownian movement of water molecules in tissues without contrast administration. Changes in the composition and/or cellularity of tissues would influence the random thermal diffusion of water molecules, which can be quantitatively measured using DWI. DWI has been used for the early diagnosis of rectal cancer and to evaluate the efficacy of neoadjuvant therapy, which may provide functional information that can be used to characterize the microstructures of tumour tissues [14]. In clinical practice, SS-EPI with a mono-exponential model remains the most commonly used method for DWI of rectal cancer. However, the optimized combination of b-values for it is still unclear. Therefore, the use of an optimized number and distribution of b-values could have a major impact on the accuracy of rectal cancer characterization, and the repeatability of DWI decay curve-derived parameters.

Modern clinical MRI scanners benefit from multiple technical advancements in radiofrequency, gradient hardware and software that have contributed to significant improvements in DWI. As such, there is an opportunity to perform multiple b-value DWI, including b-values > 2000 s/mm^2^ in the body [23]. Using multiple b-values, particularly in the range of 0–200 s/mm^2^, sensitizes the diffusion measurement to capillary perfusion and other flow phenomena [10, 11], while intermediate b-values (≥500 s/mm^2^) provide diffusion information that used for lesion characterization. However, the signal contribution of water from the extracellular space is substantially reduced at higher b-values (> 1000 s/mm^2^), making the diffusion measurement more sensitive to restrictive compartments, such as the intracellular compartment [11]. Several reports have investigated the association between the ADC value and the diagnosis of rectal cancer using ≥ 2 b-values ranging from 0 to 800-1000 s/mm^2^, but those studies didn't evaluate the effect of the b-value distribution on the diagnostic performance [12–18].

In the present study, patients with rectal cancer underwent DWI examinations using 10 b-values with a maximum b-value of 2000 s/mm^2^, employing the mono-exponential model. In theory, there are 1013 different b-value combinations. However, there were some meaningless combinations (such as two or three adjacent extremely high or low b-values) that we did not evaluated. Therefore, 31 representative combinations in total were selected with different numbers and distributions. Our results showed that the number of b-values and distributions influenced the repeatability of the ADC values and the diagnostic performance. All the b-value combinations have similar ICC values, while the bias and CV values varied and mainly affect the reproducibility of those combinations.

In this study, all the b-value distributions indicated that the mean ADC values were negatively correlated with pathological T stages of rectal cancer (all ρ values are < 0). This could be partially explained by the fact that ADC values are derived from the diffusive movement of water molecules, which is often influenced by microstructure, cell density and heterogeneity [9]. Meanwhile, higher T stage tumours showed greater heterogeneity of cell morphology and histology, higher cell density, smaller interstitium, and a lower ADC value, which could support the results of the present study. Stage T1 and T2 lesions were differentiated from T3 lesions by identification of a smooth outer tumour border within the rectal wall, with no invasion into the fat surrounding the rectum. All the ROC curves showed large AUC (> 0.7), suggesting that DWI with multiple b-values can be used to distinguish T3 lesions from T1–2 lesions and predict the behaviour of rectal cancer.

Increasing the number of b-values lead to an improvement in terms of repeatability except in the T stage. However, certain optimized b-value distributions still demonstrated an improvement in the diagnostic performance based on AUC and ρ values, such as b-values of 0 and 1000 s/mm^2^, which are the optimized combination for the reliability and diagnostic performance in the present study. In addition, it is the most commonly used b-value distribution for abdomen DWI in clinical practice [24]. Significant suppression of normal tissue can be achieved by using an ultra-high b-value (2000 s/mm^2^), providing improved tumour conspicuity and localization, but resulting in decreased SNR and an increased deformation with higher b-values. To obtain a better image quality, b = 1000 s/mm^2^ was suggested as the optimal b-value for displaying a clearer border between the tumour and normal tissues, which is helpful and useful for clinical work.

There are some limitations in this study. First, with the small number of patients, there were no T4 lesions, because we excluded patients who had received neoadjuvant therapy, but the T4 stage patients frequently received CRT. In the future, more subjects need to be recruited. Second, the acquisition time of multi-b-value DWI was relative long in the present study, it needed to have a good cooperation of patient during MRI examination, however, the scan time can be reduced using the optimal b-value combination. Third, we only used the Gaussian model to analyse data in this study, in addition, the b-factor distribution may not be optimal for non-Gaussian model, but it will be assessed in a future study.

## Conclusions

In conclusion, the number of b-values and their distributions influenced the repeatability of the ADC values and the diagnostic performance. The optimal b-value combination was 0 and 1000 s/mm^2^ for DWI examination of rectal cancer patients.

## Additional file

Additional file 1: **Table S1.** ROC analysis of the ADC parameter. (DOCX 17 kb)

## Abbreviations

ADC: Apparent diffusion coefficient
AUC: Area under receiver operating characteristic curve
CV: Coefficient of variability
DWI: Diffusion-weighted imaging
FOV: Field of view
ICC: Intraclass coefficient
MRI: Magnetic resonance imaging
ROI: Region of interest
SNR: Signal-to-noise ratio
SS-EPI: Single-shot echo-planar imaging
TR/TE: Repetition time/echo time
ρ: Spearman correlation coefficient
