# Supplementary material for: Diffusion-weighted imaging of rectal cancer on repeatability and cancer characterization: an effect of b-value distribution study
Source: Cancer Imaging. 2018 Nov 15;18:43. doi: 10.1186/s40644-018-0177-1 (PMC6238345; doi:10.1186/s40644-018-0177-1)
Supplement: Supplementary file 1 — Table S1. ROC analysis of the ADC parameter. (DOCX 17 kb) [file 40644_2018_177_MOESM1_ESM.docx]

**Supporting Table 1.** ROC analysis of the ADC parameter.

| No | b-value distribution | AUC  (95% CI) | Cutoff value  (×10^-3^ mm^2^/s) | Sensitivity  (%) | Specificity  (%) | *p* |
| --- | --- | --- | --- | --- | --- | --- |
| 2 | 0,1000 | 0.901 (0.743 - 0.978) | 0.979 | 88.9 | 85.7 | <0.0001 |
| 2 | 0,1500 | 0.829 (0.655 - 0.939) | 0.854 | 66.7 | 85.7 | <0.0001 |
| 2 | 0,2000 | 0.837 (0.664 - 0.943) | 0.761 | 66.7 | 92.9 | <0.0001 |
| 2 | 50,1000 | 0.877 (0.713 - 0.966) | 0.910 | 83.3 | 85.7 | <0.0001 |
| 2 | 50,1500 | 0.813 (0.637 - 0.929) | 0.940 | 94.4 | 57.1 | <0.0001 |
| 2 | 100,1000 | 0.730 (0.545 - 0.871) | 0.783 | 38.9 | 100.0 | 0.0100 |
| 2 | 100,2000 | 0.837 (0.664 - 0.943) | 0.796 | 100.0 | 57.1 | <0.0001 |
| 2 | 200,1000 | 0.734 (0.549 - 0.874) | 0.793 | 55.6 | 92.9 | 0.0094 |
| 2 | 200,2000 | 0.766(0.583 - 0.897) | 0.652 | 50.0 | 92.9 | 0.0018 |
| 3 | 0,100,1000 | 0.825(0.651 - 0.936) | 0.989 | 94.4 | 64.3 | <0.0001 |
| 3 | 0,200,2000 | 0.766(0.583 - 0.897) | 0.834 | 83.3 | 71.4 | 0.0022 |
| 3 | 50,1000,2000 | 0.865(0.698 - 0.959) | 0.737 | 88.9 | 71.4 | <0.0001 |
| 3 | 100,1000,1500 | 0.933(0.785 - 0.991) | 0.816 | 94.4 | 85.7 | <0.0001 |
| 3 | 200,500,1500 | 0.802(0.623 - 0.921) | 0.655 | 55.6 | 100.0 | 0.0002 |
| 3 | 500,1500,2000 | 0.937(0.791 - 0.992) | 0.644 | 88.9 | 85.7 | <0.0001 |
| 4 | 0,100,1000,1500 | 0.750(0.549 - 0.874) | 0.979 | 100.0 | 50.0 | 0.0130 |
| 4 | 0,200,1000,2000 | 0.833(0.660 - 0.941) | 0.745 | 66.7 | 85.7 | <0.0001 |
| 4 | 0,500,1000,1500 | 0.829(0.655 - 0.939) | 0.756 | 66.7 | 92.9 | <0.0001 |
| 4 | 100,200,500,1000 | 0.804(0.625 - 0.922) | 0.889 | 83.3 | 71.4 | 0.0010 |
| 4 | 500,1000,1500, 2000 | 0.938(0.793 - 0.993) | 0.601 | 72.2 | 100.0 | <0.0001 |
| 5 | 0,50,200,500,1000 | 0.714(0.528 - 0.859) | 0.944 | 72.2 | 78.6 | 0.0271 |
| 5 | 0,200,500,1000,1500 | 0.774(0.592 - 0.902) | 0.834 | 66.7 | 78.6 | 0.0010 |
| 5 | 0,500,1000,1500,2000 | 0.871(0.705 - 0.963) | 0.752 | 83.3 | 78.6 | <0.0001 |
| 5 | 50,200,500,1500,2000 | 0.865(0.698 - 0.959) | 0.721 | 77.8 | 78.6 | <0.0001 |
| 5 | 100,200,500,1000,2000 | 0.861(0.693 - 0.957) | 0.705 | 83.3 | 78.6 | <0.0001 |
| 6 | 0,100,200,500,1000,1500 | 0.829 (0.655 - 0.939) | 0.848 | 83.3 | 71.4 | <0.0001 |
| 6 | 50,200,500,1000,1500,2000 | 0.825(0.651 - 0.936) | 0.730 | 66.7 | 85.7 | <0.0001 |
| 7 | 0,100,200,500,1000,1500,2000 | 0.821(0.646 - 0.934) | 0.835 | 100.0 | 57.1 | <0.0001 |
| 8 | 0,50,100,200,250,500,1000,1500 | 0.817(0.641 - 0.931) | 0.843 | 83.3 | 78.6 | <0.0001 |
| 9 | 0,50,100,150,200,500,1000,1500,2000 | 0.857(0.688 - 0.955) | 0.757 | 72.2 | 85.7 | <0.0001 |
| 10 | All b values | 0.774(0.592 - 0.902) | 0.846 | 100.0 | 50.0 | 0.0013 |

AUC: Area under receiver operating characteristic curve, CI: Confidence interval.
